# *Punica granatum* peel supplementation attenuates cognitive deficits and brain injury in rat by targeting the Nrf2‐HO‐1 pathway

**DOI:** 10.1002/fsn3.3049

**Published:** 2022-09-12

**Authors:** Mahsan Akbarian, Mahmoud Hosseini, Farshad Mirzavi, Sabiheh Amirahmadi, Fahimeh Lavi Arab, Arezoo Rajabian

**Affiliations:** ^1^ Applied Biomedical Research Center Mashhad University of Medical Sciences Mashhad Iran; ^2^ Psychiatry and Behavioral Sciences Research Center Mashhad University of Medical Sciences Mashhad Iran; ^3^ Department of Biochemistry and Nutrition, School of Medicine Gonabad University of Medical Sciences Gonabad Iran; ^4^ Department of Immunology, Faculty of Medicine Mashhad University of Medical Sciences Mashhad Iran; ^5^ Department of Internal Medicine, Faculty of Medicine Mashhad University of Medical Sciences Mashhad Iran

**Keywords:** acetylcholinesterase, behavior performance, memory impairment, *Punica granatum* fruit

## Abstract

The critical role of nutrition to prevent neurodegenerative disorders is well documented. *Punica granatum* fruit is identified as a highly nutritional food for alleviating various ailments. The ameliorating properties of *P. granatum* peel on memory dysfunction and the possible roles of oxidative stress, acetylcholinesterase (AchE), and nuclear factor erythroid 2‐related factor 2 (Nrf2)‐heme oxygenase (HO)‐1 pathway in the scopolamine‐treated rats were assessed. The hydroethanolic extract was standardized using high‐performance liquid chromatography (HPLC). The animal groups were as follows: Control, scopolamine (2 mg/kg), and treatment groups (the extract at doses of 200–800 mg/kg). The behavioral performance was evaluated using the Morris water maze (MWM) and passive avoidance equipment. Various biochemical parameters were then measured. Rats received the extract properly found on the platform location, indicated by a shorter traveling time and distance during 5 days of learning MWM. Moreover, the extract increased the delay and light time, while decreasing dark time and the frequency of entries to the dark in the passive avoidance test. The extract also exerted a significant increase in superoxide dismutase activity and thiol content, while decreasing AchE activity and lipid peroxidation in the brain of scopolamine‐injured rats. Our results demonstrated the neuroprotective effects of *P. granatum* peel in minimizing scopolamine injury possibly through targeting the Nrf2‐HO‐1 pathway.

## Graphical abstract

The hydro‐ethanolic extract of P. granatum peel was standardized. ;The extract increased superoxide dismutase activity and thiol content, while reduced function of AchE and peroxidation of lipid in the scopolamine‐injured rat’s brain. ;P. granatum may attenuate scopolamine injury through targeting nuclear factor‐E2‐related factor 2 pathway ;

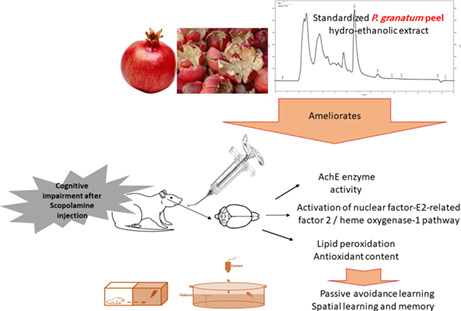

## 1 INTRODUCTION

Alzheimer's disease (AD) is associated with progressive cognitive loss and is connected to alterations in neurochemical patterns and chronic neuro‐inflammatory events. Cholinergic neurotransmission has an important role in learning, memory, and cognition. Acetylcholinesterase (AchE), as an acetylcholine (Ach)‐hydrolyzing enzyme, plays a key role in cholinergic function (Lee et al., 2017). However, aberrant AchE activity following neuronal injury potently contributes to cholinergic disturbances observed in AD (Tang, 2019). The drugs currently used to relieve symptoms of AD include donepezil, rivastigmine, memantine, tacrine, and galanthamine (Tang, 2019). Regarding the comparably low antioxidant capacity of brain tissue, an imbalance of formation and accumulation of reactive oxygen species (ROS) promotes oxidative stress (Chen & Zhong, 2014). When nuclear factor‐E2‐related factor 2 (Nrf2) is translocated into the nucleus in response to brain damage, it promotes antioxidative defense by boosting gene production of antioxidant mediators such as superoxide dismutase enzyme (SOD) and heme oxygenase‐1 (HO‐1) (Zhang et al., 2017). The antioxidant agents may have neuroprotective potential against oxidative stress in AD patients. As evident from the recent investigations, the intake of dietary antioxidants is suggested to rescue cognitive impairment (Scarmeas et al., 2018; Zhou et al., 2020).

*Punica granatum* L. (pomegranate) is used as herbal medicine and it abundantly grows in the Middle East. The main pharmacological compounds of *P. granatum* are flavonoid and phenolic compounds (like ellagic acid) that known as potent antioxidants (Sreekumar et al., 2014). Different parts of pomegranate are traditionally used in many cultures because of their beneficial effects on health (Pirzadeh et al., 2021). Various pharmacological effects including neuroprotective, anticancer, and anti‐inflammatory are reported for pomegranate (Sreekumar et al., 2014).

As shown in the literature, *P. granatum* peel and ellagic acid were found to attenuate neurodegeneration in the animal models of AD. In this context, recent studies have proposed the neuroprotective and memory‐enhancing effects of a standardized extract of pomegranate peel and ellagic acid in aluminum chloride (AlCl_3_)‐induced AD memory deficit (Harakeh et al., 2020; Ramadan & Alkarim, 2021). Consistently, Farbood et al. (2019) showed the beneficial advantages of ellagic acid on anxiety/depression‐like behaviors and cognitive deterioration in diabetic rats, as a result of regulating blood glucose levels, inflammatory state, and neurotrophic factors (Farbood et al., 2019). In separate research, pomegranate peel improved spatial memory in a mice model of AD induced by amyloid‐β (Aβ) peptide (Morzelle et al., 2016). However, it is unclear whether improvement of cholinergic dysfunction and oxidative injury contributes to the positive effects of a standardized hydroalcoholic extract of *P. granatum* peel (PPE) on the cognitive decline following scopolamine administration. Scopolamine is known as one of the nonselective antagonism of the muscarinic receptor which damages the antioxidant pathway and promotes oxidative neuronal injury (Tang, 2019). Hence, this study aimed to investigate the effects of PPE on memory function, AchE activity, oxidative stress status, and Nrf2‐HO‐1 expression in a rat model of AD‐like mediated by scopolamine.

## 2 METHODS

### 2.1 Preparing the extract

*P. granatum* was purchased from Bajestan city (Khorasan Province, Iran). The peels were collected and dried in a dark room. They were then grounded using a mortar. Following that, an appropriate amount of water‐diluted ethanol (70%) was mixed with the provided powder. The solution was shaken three times a day for 2 days. Finally, the hydroalcoholic extract was filtered and then concentrated using a rotary evaporator at 37°C. The resultant extract was stored at ‐20°C until performing the experiments (Rajabian et al., 2018). The yielded extract was 50% (w/w) of the dried powder.

### 2.2 Characterizing the peel extract

In order to quantitatively characterize *P. granatum* extract, a HPLC (high‐performance liquid chromatography) system (Berlin, Germany) was used. The chromatographic measurements were performed on a reverse‐phase water C18 analytical column (10 × 4.6 mm) with a particle size of 5 mm. A methanol–water solution (20%–100% v/v) was also used as a mobile phase. The injection volume and run time were 20 μl and 1 ml/min, respectively. The wavelength of UV detection was 254 nm.

### 2.3 Drug administration and experimental protocol

In the current research, 50 male (aged 8–10 weeks) Wistar rats (weighted 220 ± 20 g) were used. The animals were housed under the standard conditions (12 h light–dark cycles and controlled temperature). The rat had free access to standard food and water ad libitum. The guidelines provided by the National Institutes for the Care and Use of Laboratory Animals were followed during our experiments and the Animal Ethical Committee at Mashhad Medical University approved these procedures (Ethical code. IR.MUMS.MEDICAL.REC.1399.606).

The studied rats were treated according to the following protocol (10 animals per group): The experiments lasted 3 weeks. During the first 2 weeks, the control group (group I) received vehicle (tap water) at a dose of 1 ml/kg/day (oral gavage), scopolamine group (group II) received vehicle (tap water) at a dose of 1 ml/kg/day (oral gavage), and the treatment groups (groups III–V; Scopolamine‐Extract 200, Scopolamine‐Extract 400, and Scopolamine‐Extract 800) were given 200, 400 or 800 mg/kg of PPE, respectively (oral gavage). Accordingly, scopolamine was not injected during these 2 weeks. The doses of PPE administration were selected based on previous studies (Akbarian et al., 2022; Sarkaki et al., 2015; Belkacem et al., 2010). In the third week, groups I and II received the vehicle, and the treatment groups (III, IV, and V) received 200, 400, and 800 mg/kg of PPE 1 h before the behavior tests. Then, scopolamine was injected (into groups II–V at a single dose of 2 mg/kg, intraperitoneal) 30 min before the behavior tests. The saline, instead of scopolamine, was injected into group I. Finally, the behavioral tests were done 30 min after scopolamine administration (Lee et al., 2021; Sohn et al., 2021).

### 2.4 Assessment of spatial memory

Spatial memory was examined using the MWM apparatus. It included a water‐filled pool (136 cm in diameter, height of 60 cm, and depth of 30 cm) in which the temperature was kept between 22 and 24°C. The pool consists of four imaginary sections, including the north, south, east, and west section. There was a submerged circular platform 2 cm under the surface of the water in the center of the southwest area of the pool. The platform was not visible to the animals. However, there were some visual signs placed on the walls around the maze that helped the animals memorize the signs and discover the position of the platform. For each rat, 5 days experiment was done, and each experiment included four trials. For performing the trials, the rat was randomly placed into each one of the four starting positions (north, east, south, and west). It was allowed to swim for 1 min to self‐discover the location of the platform and stay on it for 15 s. If the rat did not find the platform within 1 min, it was located on the circular platform by the experimenter and was permitted to locate there for 15 s. After each trial, the rat was transferred to the cage. They had a 20 s rest period between the trails. On the 6th day, the rat was allowed to swim inside the pool for 60 s while the hidden platform had been removed (spatial probe test). The movements of the rats were recorded using a computer‐connected camera mounted above the pool. The recorded movements were then assessed using appropriate software (Radiab, Iran) and the data were collected. Information such as spent time and distance to locate the platform was recorded over the course of 5 days of training. On day 6, the data included the length of time and distance in the destination part (where the platform had been) (Lee et al., 2021; Sohn et al., 2021).

### 2.5 Passive avoidance test

An apparatus used for this test was an acrylic shuttle box which included two compartments. Each compartment had standard dimensions (30 × 20 × 20 cm). The apparatus consists of a white illuminated compartment and a dark one that was connected to each other via a small gate. The floor of both compartments contained stainless steel bars and there was a 10 mm space between the bars. The bars of the black compartment were connected to a shock generator. The experiment included three phases. In the first phase, each rat was familiarized with the apparatus. This phase lasted two consecutive days, the gate between the two compartments was opened and the rat was permitted to search the apparatus for 5 min each day. In the next (acquisition) phase, the gate was closed and each rat was gently placed inside the illuminated compartment, the gate was opened after 20 s, and the rat entered the black compartment. After closing the gate, the rat received an electrical foot shock delivered through the grid floor (2 mA, 50 Hz, 2 s). The gate was then opened and the rat moved to the illuminated compartment and, finally, transferred to the cage. In the third (retention) phase, the test was repeated 3, 48, and 72 h after the shock. In this phase, we examined whether the animals remember the shock, 3, 48, and 72 h after receiving the shock. For this purpose, the small gate was closed, and the rat was placed inside the illuminated compartment. After 20 s, the gate was opened. The step‐through latency and the time the rat spent in each black and white compartment during 300 sec were noted. The frequency of entries to the black compartment during 300 sec was also noted (Lee et al., 2021; Sohn et al., 2021).

### 2.6 The biochemical measurements

The rats were sacrificed immediately following the behavioral tests, and the whole‐brain tissues were rapidly separated. In ice‐cold conditions, the cortex and hippocampus of the brain were removed and cleaned with ice‐cold normal saline. A tissue homogenate [10% (w/v)] was prepared in sodium phosphate buffer (PBS) and centrifuged at 1500 revolutions per minute (rpm) for 10 min at 4°C, and the supernatant was used for estimation of oxidative stress markers.

### 2.7 Estimation of lipid peroxidation

In this experiment, malondialdehyde (MDA) was estimated as an indicator of lipid peroxidation. For this purpose, 1 ml of each sample (supernatant) was mixed with 2 ml of working solution (containing 15% (w/v) trichloroacetic acid, 0.37% (w/v) thiobarbituric acid, and 2% (v/v) hydrochloric acid) and the tubes were kept in a boiling water bath for 45 min. Following that, the tubes were cooled and centrifuged at 1500 rpm for 10 min. Finally, the supernatant absorbance was read by a spectrophotometer at a wavelength of 535 nm. The result was expressed as nmol/g tissue (Hosseini et al., 2022).

$$\text{Concentration}\;\left(\mathrm{mol}/L\right)=\text{Absorbance}/1.65\times 10^{5}$$

### 2.8 Estimation of total thiol content

The total thiol content (or sulfhydryl groups) in the brain tissue was detected using 5,5′‐dithiobis (2‐nitrobenzoic acid) (DTNB), as a reagent (Hosseini et al., 2022). DTNB reacts with sulfhydryl groups, and a Uv–vis spectrophotometer detects the absorbance. For this purpose, 50 μl of each sample was mixed with 1 ml of tris‐ethylenediaminetetraacetic acid (EDTA) buffer (pH = 8.26) and the absorbance was detected (A1) at 412 nm. The provided DTNB reagent (20 μl, 10 mmol/L) was then dispensed into the tubes, and the second absorbance (A2) was read at the same wavelength. The absorbance of the DTNB reagent was also measured (B). To calculate total thiol content, the following equation was used.

$$\text{Total thiol concentration}\;\left(\text{mmol}/l\right)=\left(\text{A2}-\text{A1}-B\right)\times 1.07/\left(0.05\times 13.6\right).$$

### 2.9 Estimation of SOD activity

Superoxide dismutase activity in the brain tissue was measured by the method defined by Madesh and Balasubramanian (Madesh & Balasubramanian, 1998). The method is based on the ability of SOD enzyme to scavenge free radicals produced by pyrogallol autoxidation. For this purpose, 60 μl of each sample was dispensed into each well of a 96‐well plate containing PBS (60 μl). Following that, 15 μl of pyrogallol solution (0.1 mg/ml) and MTT (3‐[4,5‐dimethylthiazol‐2‐yl]‐2,5‐diphenyltetrazolium) 6 μl, 0.5 mg/ml were added. The plate was then incubated for 5 min at room temperature. Finally, dimethyl sulfoxide was added to solubilize the produced color. The optical absorbance was measured at 570 nm. The brain tissue SOD activity was expressed as units per gram of tissue (Hosseini et al., 2022).

### 2.10 Evaluation of AchE enzyme level

The AchE activity in the brain was estimated according to an assay set by Ellman (Ellman et al., 1961). The principle of this assay is based on the rate of thiocholine formation from acetylthiocholine iodide in the presence of the AchE enzyme, which was recorded by a spectrophotometer at 412 nm. For this purpose, the tissue homogenate (40 μl) was dispensed into the tubes containing cold PBS (3 ml). One hundred microliterl of DTNB (0.01 mol/L) was transferred to each tube, then vortexed and kept for 10 min at room temperature. Following that, acetylthiocholine iodide (0.07 mol/L, 20 μl) was added to start the reaction and the absorbance was read. After 10 min, the second absorbance was read and the change in absorbance was calculated. AchE activity was estimated by the following formula and expressed as nmol/min/tissue (g) (Hosseini et al., 2022; Ugbaja et al., 2021).

$$R=5.74\;\left(10^{-4}\right)\;\mathrm{\Delta A}/C_{0}$$

where R = rate in moles substrate hydrolyzed per min per tissue (g); ΔA = change in absorbance per min; and C_0_ = original concentration of tissue.

### 2.11 Quantitative real‐time polymerase chain reaction (RT‐PCR) analysis

To detect messenger RNA (mRNA) expression of Nrf2 and HO‐1, quantitativeRT‐PCR test was performed based on SYBER green technology. Table 1 shows the forward and reverse sequences of primers. Total RNA was isolated from tissue homogenates of the hippocampus and RNA levels were measured using NanoDrop 1000 (Thermo). Then, the RNA quality was evaluated by the PCR method. Extracted RNA was subjected to complementary DNA (cDNA) synthesis, and the first‐strand cDNA was generated using reverse transcription of relevant mRNAs according to the instructions of the Easy cDNA Synthesis kit (Pars Tous) and SYBR Green real‐time PCR kit (Pars Tous). Real‐time method was performed by LightCycler® 96 RT‐PCR system (Roche). Mean relative quantification of cycle threshold (CT) was calculated with normalization to glyceraldehyde‐3‐phosphate dehydrogenase (GAPDH) level as the internal control reference gene. The comparative Ct method (ΔΔCt) was used for RT‐PCR data analysis. Data are presented as fold change relative to control. The formula of 2^−ΔΔCt^ measured the relative gene expression of the target genes (Ugbaja et al., 2021).

$${\mathrm{\Delta Ct}}_{\text{target}}={\mathrm{Ct}}_{\text{target}}-{\mathrm{Ct}}_{\text{internal control}}$$

$$\text{ΔΔCt}={\mathrm{\Delta Ct}}_{\text{target}}-{\mathrm{\Delta Ct}}_{\text{control}}\;\text{Fold change}=2^{-\text{ΔΔCt}}$$

**TABLE 1:** Primer sequences of Nrf2, HO‐1, and GAPDH

Gene name	Forward (5′‐ > 3′)	Reverse (5′‐ > 3′)	Product length (bp)
Nrf2	CTGTCAGCTACTCCCAGGTTG	AAGCGACTCATGGTCATCTACA	113
HO‐1	ATCGTGCTCGCATGAACACT	AGCTCCTCAAACAGCTCAATGT	106
GAPDH	AGTGCCAGCCTCGTCTCATA	TGAACTTGCCGTGGGTAGAG	189

### 2.12 Statistical analysis

In this research, data analysis was performed using SPSS software. For this purpose, repeated measures and one‐way analysis of variance (ANOVA), as well as Bonferroni and Tukey post hoc tests were used. Mean ± standard error of the mean (SEM) value was reported and the lowest level of significance was a *p* value less than .05.

## 3 RESULTS

### 3.1 HPLC analysis

**FIGURE 1:**
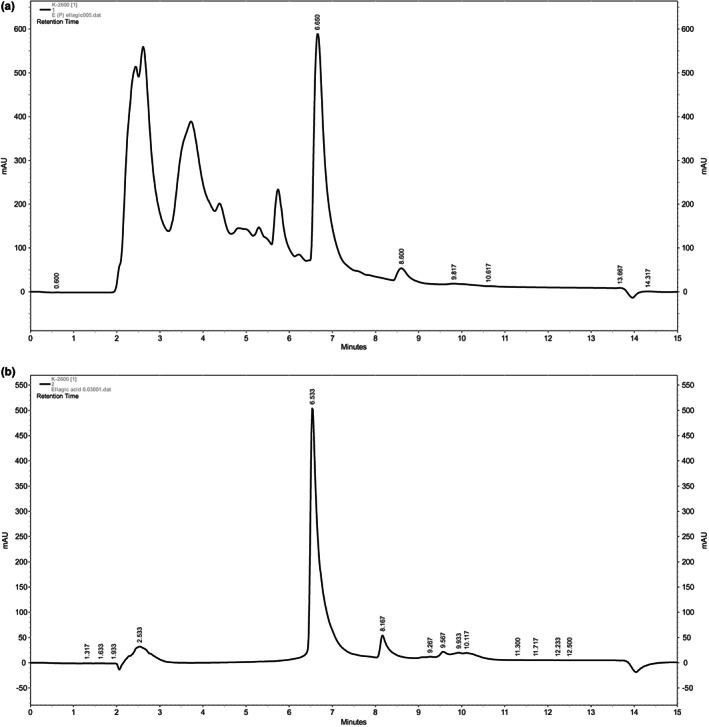
Chromatographic measurements of (a) *P. granatum* peel hydroethanolic extract (b) ellagic acid (with a specific retention time of 6.7 min). HPLC was performed on a reverse‐phase waters C18 analytical column (10 × 4.6 mm, 5 mm particle size). The chromatographic conditions include a mixture of methanol/deionized water (20/100, v/v) for elution, the flow rate of 15 min, and the wavelength of detection of 254 nm

The chromatographic pattern of the extract was compared to that of the retention time of ellagic acid, as a standard. The amount of ellagic acid estimated in the extract (g) was 1.7% (Figures 1a,b). As shown in Figure 1b, specific retention time detected for ellagic acid was 6.7 min.

### 3.2 Effect of PPE on MWM performance

**FIGURE 2:**
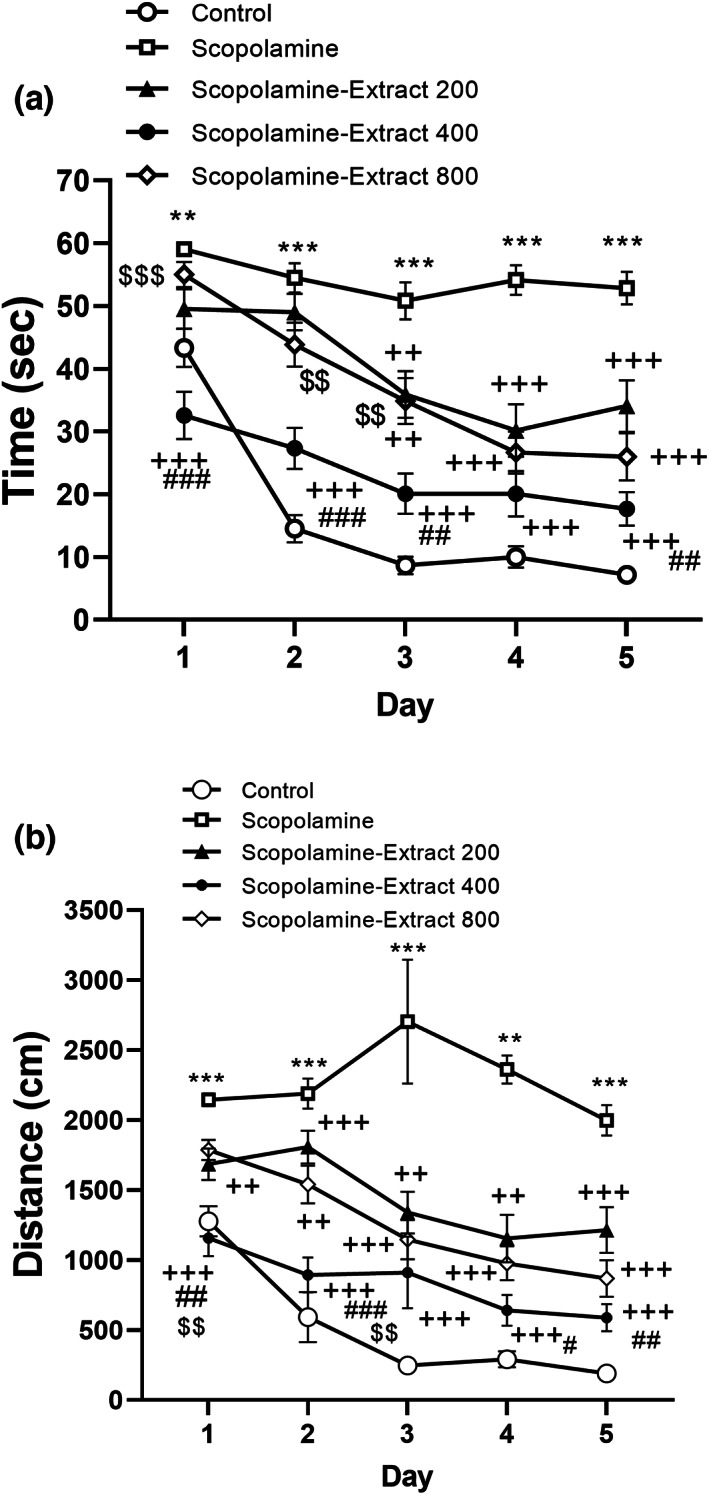
*P. granatum* peel extract improved the performance of the rats during learning phase of the Morris water maze. The rats received the extract (200, 400, and 800 mg/kg) or vehicle for 3 weeks. In the 3rd week, scopolamine was administered before the behavioral task. Treatment with the extract shortened traveling time (a) and distance (b) to find the platform. Data are presented as mean ± SEM (*n* = 10). **p < .01 or ***p < .001 comparing with control, ^++^ *p* < .01 or ^+++^ *p* < .001 comparing with scopolamine, ^##^ *p* < .01 or ^###^ *p* < .001 comparing with Scopolamine‐Extract 200, and ^$$^ *p* < .01 or ^$$$^ *p* < .001 comparing with Scopolamine‐Extract 400

There was a significant main effect of treatment (*F*_(4,195)_ = 59.36, *p* < .001 for time and *F*_(4,195)_ = 57.44, *p* < .001 for distance), day (*F*_(4, 780)_ = 56.56; *p* < .001 for time and *F*_(4,780)_ = 16.79, *p* < .001 for distance), and Treatment × Day interaction (*F*_(16, 780)_ = 5.11; *p* < .001 for time and *F*_(16,780)_ = 3.15, *p* < .001 for distance) on the time and distance to find the platform. Scopolamine administration affected the MWM performance, as seen by an increase in traveling time and distance during a 5‐day period, according to the results of a Bonferroni pairwise comparison (Figure 2, *p* < .001). Moreover, all doses of PPE reversed the effect of scopolamine on MWM performance confirmed by shorter traveling time and distance in Scopolamine‐Extract 200, Scopolamine‐Extract 400, and Scopolamine‐Extract 200 groups relative to those of the scopolamine group (both variables with *p* < .01 to *p* < .001). Notably, lower traveling time and distance were observed in the Scopolamine‐Extract 400 group compared to those of the Scopolamine‐Extract 200 group (*p* < .05 to *p* < .001). A higher traveling time and distance in the Scopolamine‐Extract 800 group were also found compared to those of the Scopolamine‐Extract 400 group (*p* < .01 to *p* < .001).

**FIGURE 3:**
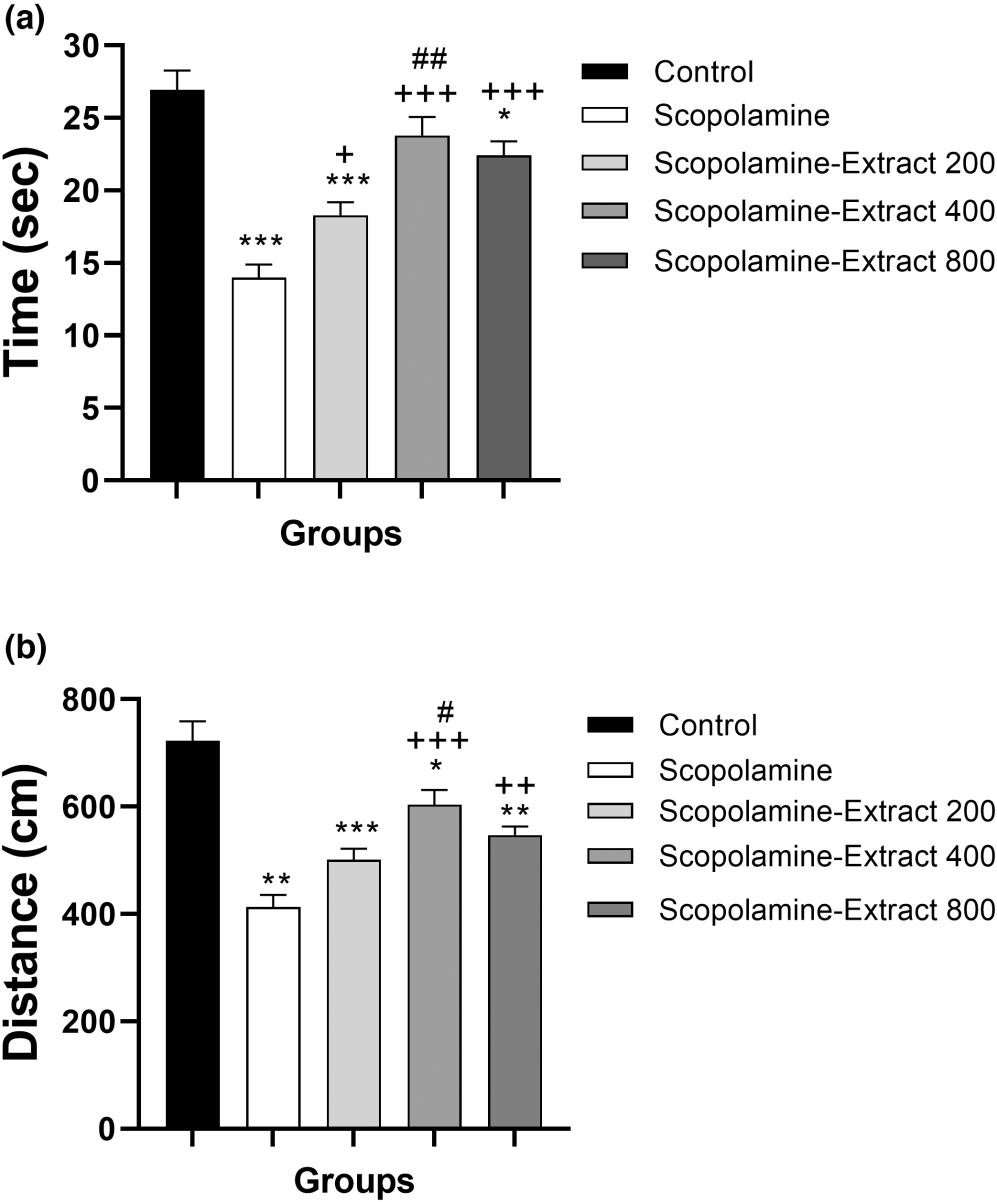
*P. granatum* peel extract improved the performance of the rats during the probe test of the Morris water maze. The rats received the extract (200, 400, and 800 mg/kg) or vehicle for 3 weeks. In the 3rd week, scopolamine was administered before the behavioral task. Treatment with the extract prolonged traveling time (a) and distance (b) in the target area. Data are expressed as mean ± SEM (*n* = 10). *p < .05, **p < .01, or ***p < .001 comparing with control, ^+^ *p* < .05, ^++^ *p* < .01, or ^+++^ *p* < .001 vs. scopolamine, and ^#^ *p* < .05 or ^##^ *p* < .01 comparing with Scopolamine‐Extract 200

The results of probe test showed that there was a significant difference among the groups in terms of traveling time and distance in the target area (*F*_(4, 195)_ = 21.10, *p* < .001, for traveling time and *F*_(4, 195)_ = 20.55, *p* < .001, for traveling distance). The probe results also revealed that scopolamine administration was accompanied by a shorter traveling time and distance in the target segment compared to the control group (Figure 3, for both variables *p* < .01 to *p* < .001). All treatment groups traveled a longer time to the destination compared to the scopolamine (*p* < .05 to *p* < .001). Furthermore, the traveling distance to the destination by both Scopolamine‐Extract 400 and Scopolamine‐Extract 800 groups was longer than that of scopolamine group (with *p* < .001 for a dose of 400 and *p* < .01 for 800). The rats of the Scopolamine‐Extract 400 group also revealed a longer traveling distance and time than those of the Scopolamine‐Extract 200 group (*p* < .05 and *p* < .01). Furthermore, the traveling time of both Scopolamine‐Extract 200 and Scopolamine‐Extract 800 groups was shorter relative to the control group (ranging from *p* < .05 to *p* < .001). In addition, all Scopolamine‐Extract 200, Scopolamine‐Extract 400, and Scopolamine‐Extract 800 groups showed shorter traveling distances relative to control group (ranging from *p* < .05 to *p* < .001).

### 3.3 Effect of PPE on PA performance

**FIGURE 4:**
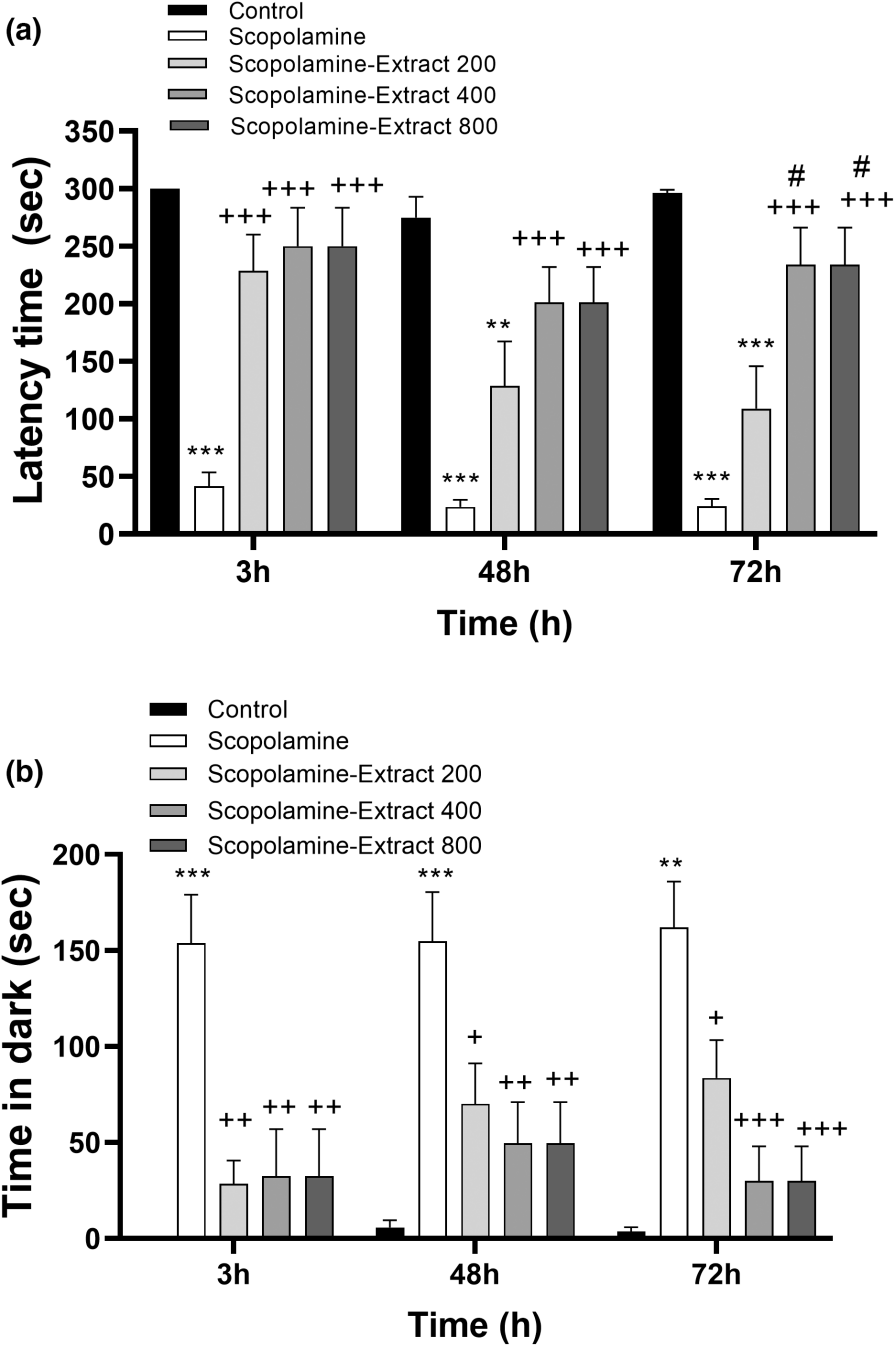
*P. granatum* peel extract improved passive avoidance performance. The rats received the extract (200, 400, and 800 mg/kg) or vehicle for 3 weeks. In the third week, scopolamine was administered before the behavioral task. Treatment with the extract prolonged the latency to enter the black area (a), while decreasing the time spent in the black area (b), 3, 48, and 72 h postshock. Data are expressed as mean ± SEM (*n* = 10). **p < .01 or ***p < .001 comparing with control, ^+^ *p* < .05, ^++^ *p* < .01, or ^+++^ *p* < .001 comparing with scopolamine, and ^#^ *p* < .05 comparing with Scopolamine‐Extract 200

The results showed that there was a significant difference among the groups in term of delay time for entrance to the black compartment, 3 h (*F*_[4, 45]_ = 14.81, *p* < .001), 48 h (*F*_[4, 45]_ = 11.99, *p* < .001), and 72 h postshock (*F*_[4, 45]_ = 17.57, *p* < .001). The behavioral results also revealed that scopolamine injection disturbed PA performance as reflected by a reduction in the delay time for entrance to the black compartment, 3, 48, and 72 h postshock (Figure 4, *p* < .001). All doses of PPE prolonged the delay time, 3 h postshock time (*p* < .001 for different doses). Moreover, PPE at both doses of 400 and 800 mg/kg prolonged delay time, 48 and 72 h postshock (*p* < .001). Moreover, the delay time in both Scopolamine‐Extract 400 and Scopolamine‐Extract 800 groups was longer than that of Scopolamine‐Extract 200 group, 72 h postshock (*p* < .05).

The results also showed a significant difference among the groups in term of total time spent in the dark, 3 h (*F*_[4, 45]_ = 9.00, *p* < .001), 48 h (*F*_[4, 45]_ = 7.40, *p* < .001), and 72 h (*F*_[4, 45]_ = 12.33, *p* < .001) postshock. Furthermore, the results revealed a longer total time spent in the dark in scopolamine group than the control group, 3, 48, and 72 h postshock (*p* < .01 for different points). The administration of PPE (at all doses) caused a significant decrease in the time spent in the dark compared to scopolamine group, 3, 24, 48, and 72 h postshock (ranging from *p* < .05 to *p* < .001 for all points).

**FIGURE 5:**
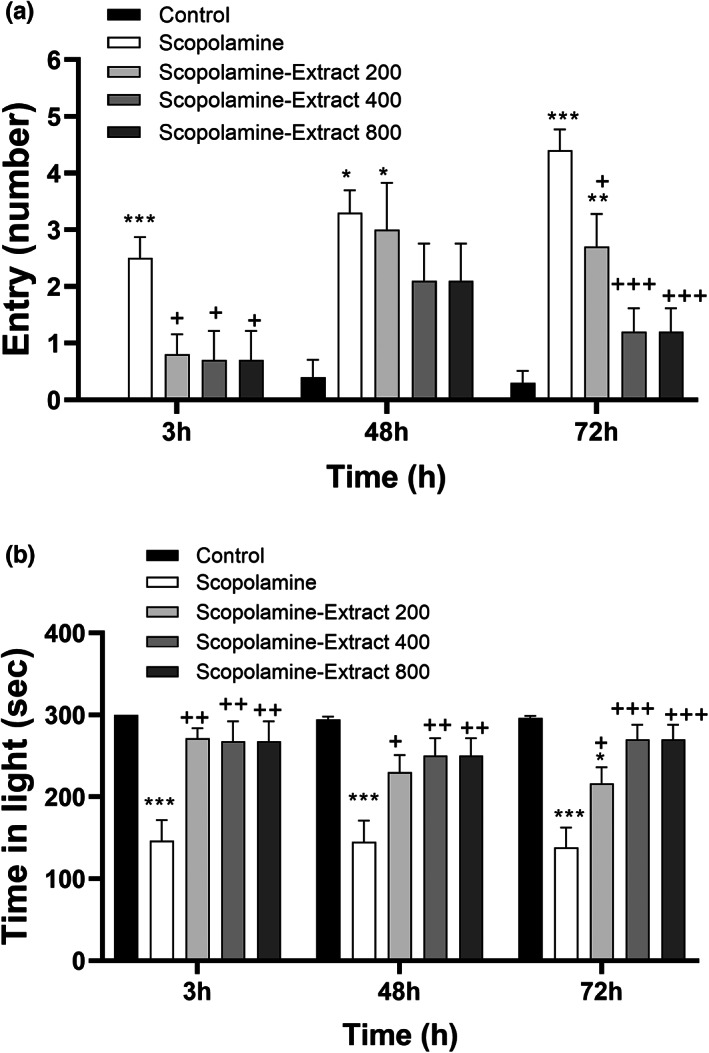
*P. granatum* peel extract improved passive avoidance performance. The rats received the extract (200, 400, and 800 mg/kg) or vehicle for 3 weeks. In the 3rd week, scopolamine was administered before the behavioral task. Treatment with the extract prolonged the time spent in the light area (a), while decreasing the entries to the black area (b), 3, 48, and 72 h postshock. Data are expressed as mean ± SEM (*n* = 10). *p < .05, **p < .01, or ***p < .001 comparing with control, and ^+^ *p* < .05, ^++^ *p* < .01, or ^+++^ *p* < .001 comparing with scopolamine group

There was also a significant difference among the groups in term of frequency of entry to the dark, 3 h (*F*_[4, 45]_ = 5.37, *p* < .001), 48 h (*F*_[4, 45]_ = 3.54, *p* < .01), and 72 h (*F*_[4, 45]_ = 15.05, *p* < .001) postshock. Scopolamine administration was also accompanied by an increase in the frequency of entry to the dark, 3, 48, and 72 after the shock (Figure 5, *p* < .001, *p* < .05, and *p* < .001, respectively). All doses of PPE decreased the frequency of entry to the dark, 3 and 72 h postshock (ranging from *p* < .05 to *p* < .001).

Our results also exhibited that there was a significant difference among the groups in term of total time spent in the light, 3 h (*F*_[4, 45]_ = 9.00, *p* < .001), 48 h (*F*_[4, 45]_ = 7.40, *p* < .001), and 72 h (*F*_[4, 45]_ = 12.18, *p* < .001) postshock. Scopolamine injection shortened the total time recorded in the light section, 3, 48, and 72 h after the punishment (Figure 5, *p* < .001 for all). In contrast, all doses of PPE increased the total light time compared to scopolamine group, 3, 48, and 72 h postshock (ranging from *p* < .05 to *p* < .001).

### 3.4 Effect of PPE on the biochemical indicators

**FIGURE 6:**
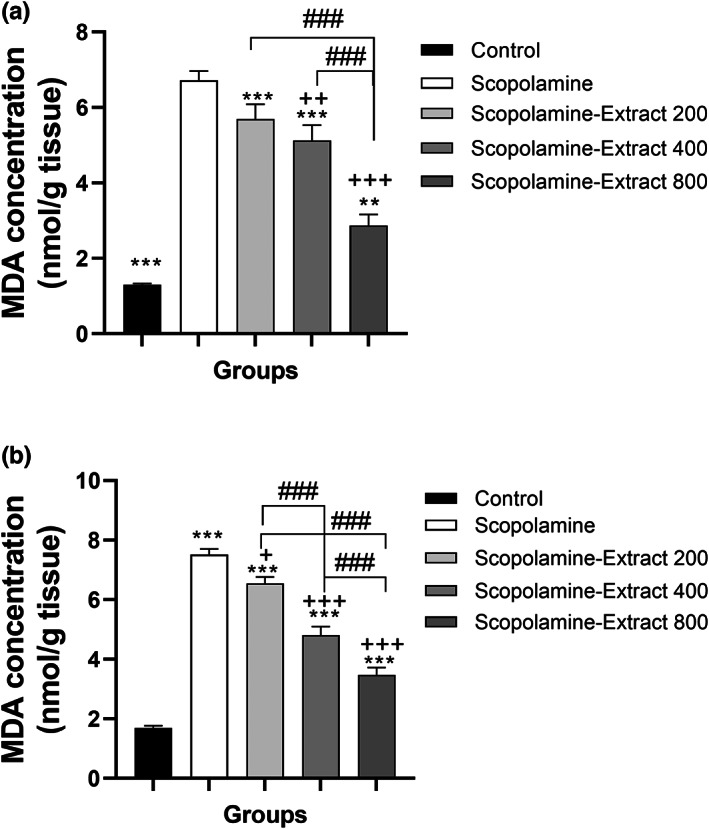
*P. granatum* peel extract modified MDA level in the cortex (a) and hippocampus (b) of the rats treated with the extract (200, 400, and 800 mg/kg, 3 weeks). For this purpose, the thiobarbituric reagent was mixed with each sample, and the absorbance of the resultant pink chromogen was measured at 535 nm. Treatment with 200, 400, and 800 mg/kg of the extract decreased MDA in both hippocampal and cortical areas. Data are expressed as mean ± SEM (*n* = 10). ***p < .01 or ***p < .001 comparing with control, ^+^ *p* < .05 or ^+++^ *p* < .001 comparing with scopolamine, and ^###^ *p* < .001 comparing with other extract‐treated groups

The results revealed a significant difference among the groups in terms of MDA concentration in the cortex and hippocampus (*F*_(4, 45)_ = 52.31, *p* < .001 and *F*_(4, 45)_ = 117.4, *p* < .001, respectively). As shown in Figure 6, scopolamine administration was accompanied by an increased MDA concentration in the hippocampus and cortex (both *p* < .001). It is notable that PPE reduced the level of MDA in the cortex of Scopolamine‐Extract 400 and Scopolamine‐Extract 800 groups relative to the scopolamine group (for both groups, *p* < .01 and *p* < .001). Moreover, MDA concentration was decreased in the hippocampus of Scopolamine‐Extract 200, Scopolamine‐Extract 400, and Scopolamine‐Extract 800 groups compared to that of scopolamine group (*p* < .05 and *p* < .001). Notably, MDA levels in the cortex and hippocampus of Scopolamine‐Extract 800 and Scopolamine‐Extract 400 groups were observed to be smaller than those of the Scopolamine‐Extract 200 group. Accordingly, the extract indicated a dose‐dependent effect on MDA (*p* < .001 for all).

**FIGURE 7:**
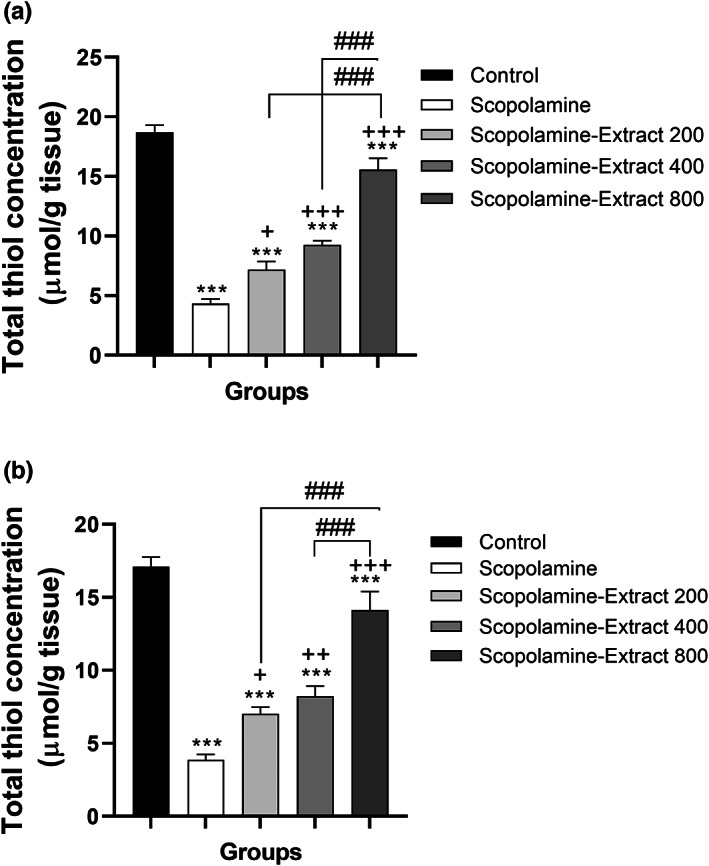
*P. granatum* peel extract modified thiol content in the cortex (a) and hippocampus (b) of the rats treated with the extract (200, 400, and 800 mg/kg, 3 weeks). This experiment was based on the reaction of the DTNB reagent with the thiol groups detected using a spectrophotometer at 412 nm. Treatment with 200, 400, and 800 mg/kg of the extract increased total thiol content in both hippocampal and cortical areas. Data are expressed as mean ± SEM (*n* = 10).***p < .001 comparing with control, ^+^ *p* < .05, ^++^ *p* < .01, or ^+++^ *p* < .001 comparing with scopolamine, and ^###^ *p* < .001 comparing with other extract‐treated groups

Our results also revealed a significant difference among the groups in terms of total thiol level in the cortex and hippocampus (*F*_(4, 45)_ = 90.97, *p* < .001 and *F*_(4, 45)_ = 51.10, *p* < .001, respectively). Scopolamine injection induced a decrement in the thiol concentration in the cortex and hippocampus compared to that of the control group (both *p* < .001, Figure 7). The thiol content in the cortex and hippocampus of all Scopolamine‐Extract 200, Scopolamine‐Extract 400, and Scopolamine‐Extract 800 groups was increased relative to those of the scopolamine group (*p* < .05 to *p* < .001). The extract exhibited a dose‐dependent effect on thiol content in the cortex and hippocampus confirmed by a higher level of the thiol content in Scopolamine‐Extract 800 and Scopolamine‐Extract 400 groups compared to that of the Scopolamine‐Extract 200 (for all groups, *p* < .001).

**FIGURE 8:**
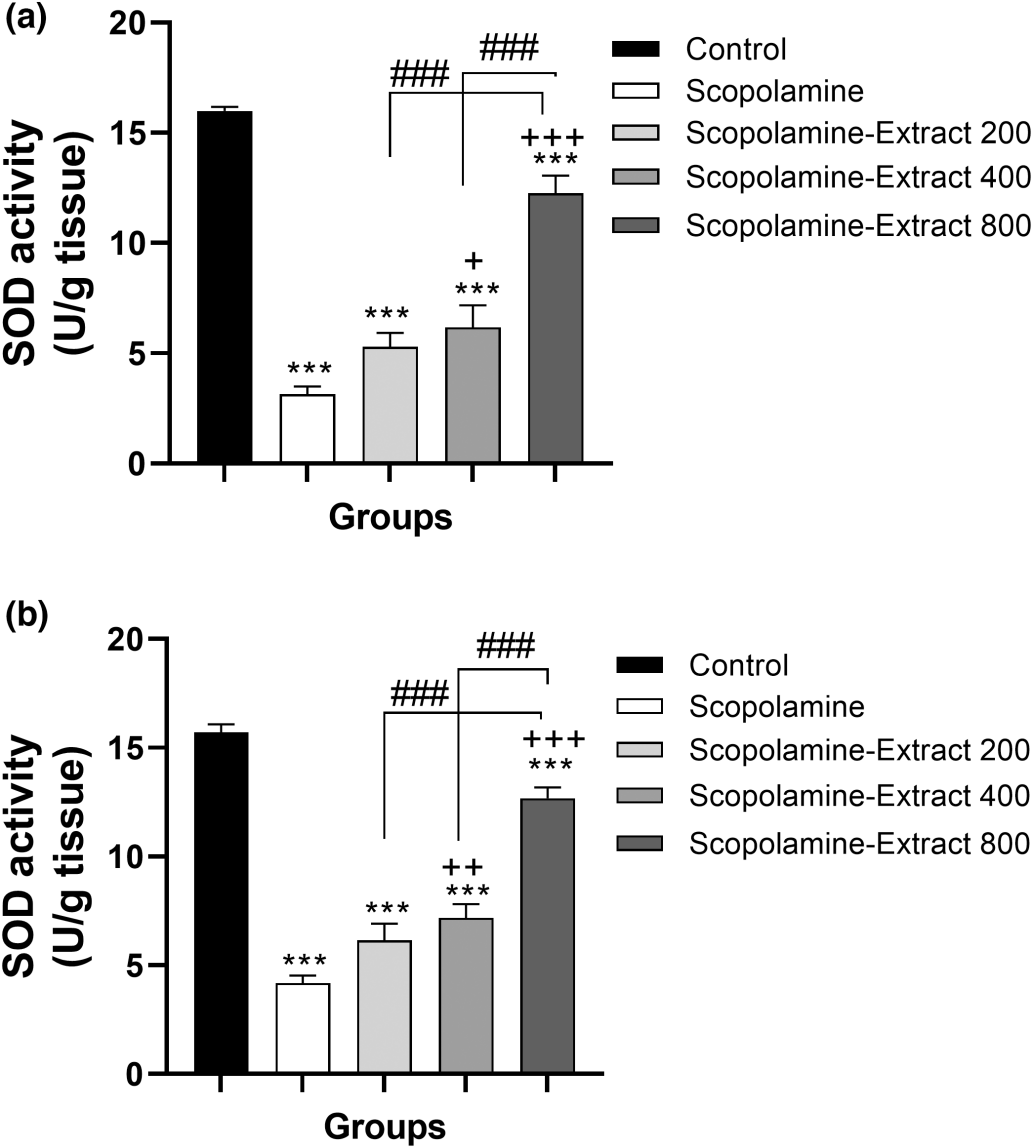
*P. granatum* peel extract modified SOD function in the cortex (a) and hippocampus (b) of the rats treated with the extract (200, 400, and 800 mg/kg, 3 weeks). For this purpose, the samples were dispensed into a solution containing MTT and pyrogallol. The absorbance was noted using an ELISA reader (570 nm). Treatment with 200, 400, and 800 mg/kg of extract increased SOD in the hippocampal and cortical sections. Data are expressed as mean ± SEM (*n* = 10). ***p < .001 comparing with control, ^+^ *p* < .05, ^++^ *p* < .01, or ^+++^ *p* < .001 comparing with scopolamine, and ^###^ *p* < .001 comparing with other extract treated groups

The oxidative stress findings also demonstrated a significant difference among the groups in terms of SOD activity in the cortex and hippocampus (*F*_(4, 45)_ = 65.11, *p* < .001 and *F*_(4, 45)_ = 79.65, *p* < .001, respectively). As illustrated in Figure 8, SOD activity in the cortex and hippocampus of the scopolamine group was lower than that of the control group (*p* < .001). SOD activity in the cortex and hippocampus of Scopolamine‐Extract 400 and Scopolamine‐Extract 800 groups was higher relative to the scopolamine group (ranging from *p* < .05 to *p* < .001). Notably, a dose‐related effect of PPE on SOD activity in both hippocampus and cortex was observed as indicated by a greater SOD activity in the Scopolamine‐Extract 800 and Scopolamine‐Extract 400 groups compared to that of Scopolamine‐Extract 200 group (*p* < .001 for all; Figure 8).

**FIGURE 9:**
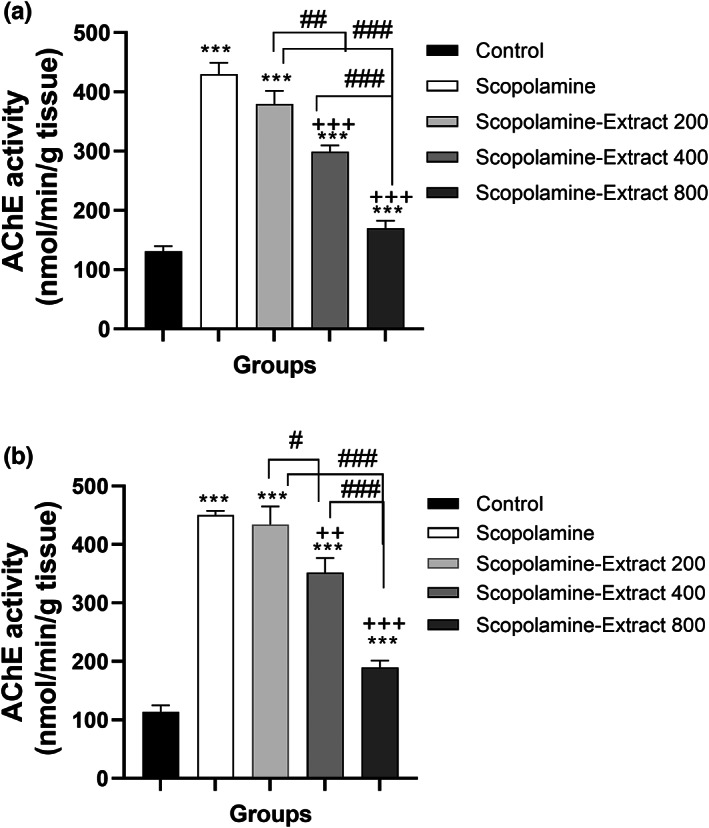
*P. granatum* peel extract modified AchE activity in the cortex (a) and hippocampus (b) of the rats treated with the extract (200, 400, and 800 mg/kg, 3 weeks). For this purpose, the changes in absorbance of each sample containing DTNB and acetylthiocholine were measured using a spectrophotometer at 412 nm. Treatment with 200, 400, and 800 mg/kg of the extract decreased AchE activity in the hippocampal and cortical sections. Data are expressed as mean ± SEM (*n* = 10). ***p < 0.001 comparing with control, ^+^ *p* < .05, ^++^ *p* < .01, or ^+++^ *p* < .001 comparing with scopolamine, and ^#^ *p* < .05, ^##^ *p* < .01, or ^###^ *p* < .001 comparing with other extract‐treated groups

There was a significant difference among the groups in terms of AchE activity in the cortex and hippocampus (*F*_(4, 45)_ = 69.87, *p* < .001 and *F*_(4, 45)_ = 59.30, *p* < .001, respectively). The cholinergic function was dysregulated following scopolamine administration which was confirmed by a decreased activity of AchE in both hippocampus and cortex (both *p* < .001). In contrast, PPE modified the AchE activity in the brain areas of the Scopolamine‐Extract 400 and Scopolamine‐Extract 800 groups compared to the scopolamine group (*p* < .05 to *p* < .001). Notably, PPE exerted a dose‐dependent effect on AchE activity which was indicated by greater activity in the cortex and hippocampus of Scopolamine‐Extract 800 and Scopolamine‐Extract 400 groups than those of the Scopolamine‐Extract 200 (*p* < .001 for all; Figure 9).

### 3.5 PPE effect on level of Nrf2 and HO‐1 mRNA

**FIGURE 10:**
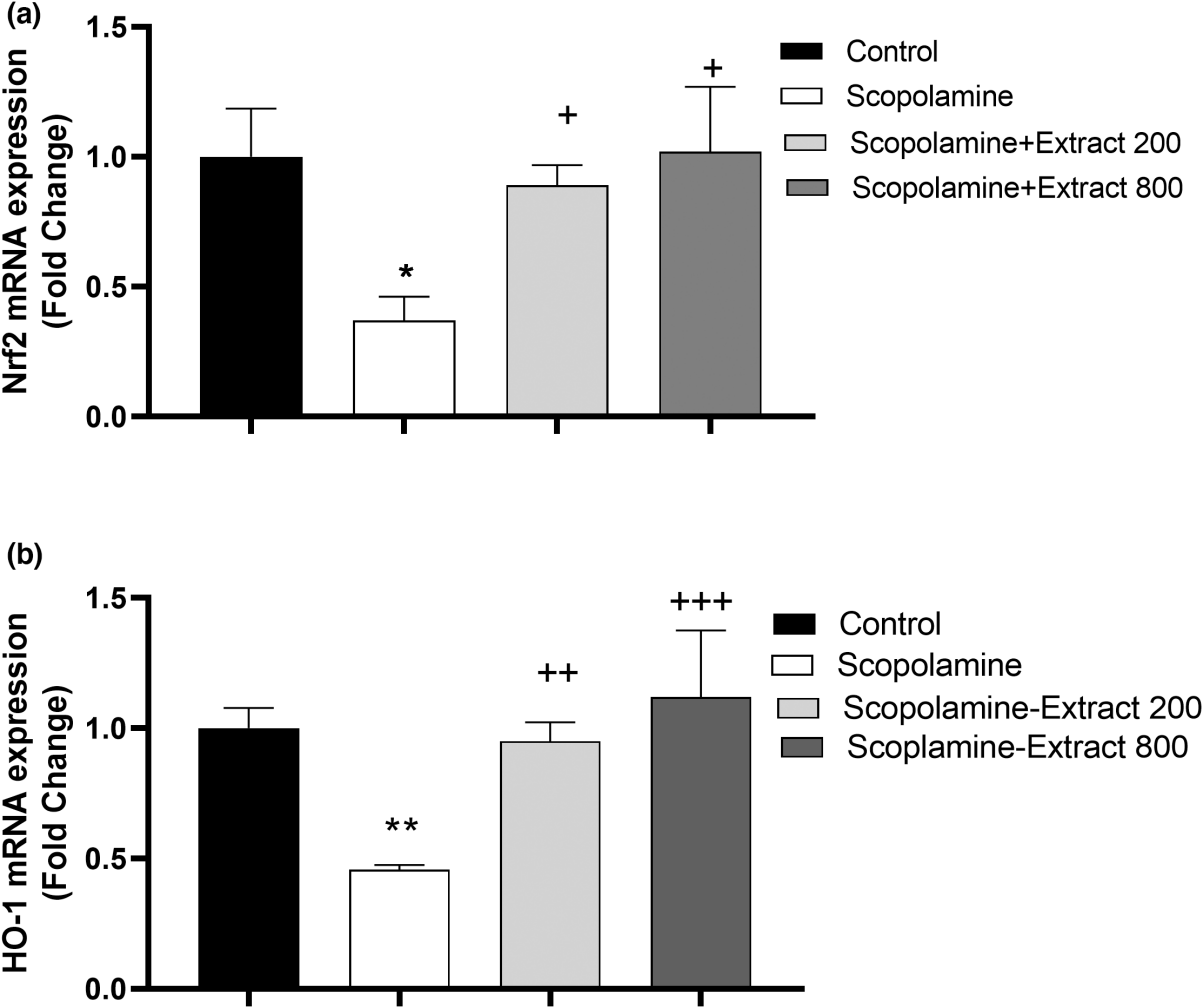
*P. granatum* peel extract‐modified mRNA levels of Nrf2 (a) HO‐1 (b) in the hippocampus. For this purpose, hippocampus‐extracted RNA was subjected to cDNA synthesis and qRT‐PCR was carried out. Fold change in mRNA expression of Nrf2 and HO‐1 was normalized to GAPDH as a reference gene. Treatment with the extract upregulated Nrf2 and HO‐1 expression in the hippocampus. Data are expressed as mean ± SEM. *p < .05 and **p < .01 comparing with control. ^+^ *p* < .05, ^++^ *p* < .01, and ^+++^ *p* < .001 comparing with scopolamine

As illustrated in Figure 10, there was a significant difference among the groups in terms of Nrf2 and HO‐1 expression in the hippocampus (*F*_(3, 12)_ = 6.39 *p* < .01 and *F*_(3, 12)_ = 11.76, *p* < .001, respectively). It is notable that the expression of HO‐1 and Nrf2 was significantly lower in the brain of scopolamine group relative to that of the control group (*p* < .05 and *p* < .001 for both variables). It was found that 200 and 800 mg/kg of PPE remarkably upregulated HO‐1 and Nrf2 mRNA expression (*p* < .05 to *p* < .001).

## 4 DISCUSSION

Our research proposed that PPE prevented memory and cognitive dysfunction in scopolamine‐injured rats by targeting the oxidative stress, Nrf2‐HO‐1 pathway, and AchE. The model of scopolamine‐mediated memory deficit is widely used to investigate the efficacy of therapeutic agents in the management of dementia (Tang, 2019). Scopolamine, a muscarinic receptor blocker agent, can cause neuronal injury and disruption of cognitive performance (Haam & Yakel, 2017; Tang, 2019). Using the scopolamine model, the pivotal role of the central cholinergic system in regulating memory and learning ability was confirmed (Haam & Yakel, 2017; Tang, 2019).

According to our results, scopolamine exerted a negative effect on learning and memory ability. The data of MWM performance during the learning period showed a longer time and distance to the destination in the rats that received scopolamine. Furthermore, the traveling time and distance were reduced in the probe test. The scopolamine‐injected rats also manifested a shortened delay time to enter the darkness in the PA test. Scopolamine also increased the time spent and frequency of entries to the dark. These findings confirmed that scopolamine affected the ability of animals to recall the punishment. Consistent with these findings, previous studies have shown that scopolamine injection prolonged escape latency and swim distance in MWM trials (Lee et al., 2021; Park et al., 2019). Furthermore, Lee et al. (2017) showed a negative effect of scopolamine on PA performance tests indicated by a shortened latency to enter darkness and light stay, while increasing the frequency of entries and dark stay time (Lee et al., 2017; Tang, 2019).

It is well known that scopolamine promotes neuroinflammation and oxidative stress, which in turn leads to memory impairment (Akbarian et al., 2022; Lee et al., 2017, 2021; Sohn et al., 2021; ). In the present study, scopolamine injection caused brain oxidative damage by reduction in the total thiol concentration, suppressing SOD activity, and enhancement of the MDA level. Consistently, Sohn et al. (2021) demonstrated a significant reduction in SOD and an increased level of MDA in the prefrontal cortex of scopolamine‐treated rats (Sohn et al., 2021). Thus, these results support the idea that the detrimental effects of scopolamine on cholinergic function were linked to oxidative damage generated in the brains of amnesic rats (Akbarian et al., 2022; Sohn et al., 2021; ). Ach is well known to have a crucial role in cholinergic functions which is essential for learning and memory performance (Lee et al., 2017; Tang, 2019). Scopolamine was found previously to increase AchE in the hippocampus and prefrontal cortex and cause memory deficits (Lee et al., 2021; Sohn et al., 2021).

The results of this study suggested that PPE administration might counteract the negative effects of scopolamine on learning and memory in rats. When investigated using the MWM test, animals in the groups treated with PPE had shorter escape latencies and distances than the scopolamine group. The results of probe trials also indicated that PPE improved memory retrieval and helped the animals recall the platform in a dose‐dependent manner. Furthermore, all doses of PPE alleviated different parameters of passive avoidance memory. In case of latency to enter the dark segment, PPE exerted a dose‐dependent effect. Previous studies reported the efficacy of pomegranate peel in the improvement of cognitive functions and neuroprotection (Adiga et al., 2010; Harakeh et al., 2020). For instance, the peel extract supplementation (100 mg/kg, 2 weeks) improved spatial learning and memory impaired by diazepam in the rat (Adiga et al., 2010). Harakeh et al. (2020) revealed that administration of pomegranate peel extract solution (50 mg/kg) and ellagic acid for 4 weeks improved memory deficit and degenerative changes in an AlCl_3_‐induced rat model of AD (Harakeh et al., 2020). Another study exhibited that pomegranate extract (100 and 200 mg/kg) exerted cognitive‐enhancing effects possibly via the depletion of Aβ aggregates in the hippocampus (Ahmed et al., 2014). Furthermore, *P. granatum* juice (500 mg/kg oral) was shown to ameliorate passive avoidance response in scopolamine‐mediated cognition dysfunction (Fatima et al., 2017).

To have an insight into the mechanisms of memory‐enhancing effects of PPE, oxidative stress indicators and AchE activity were measured in the present study. Our results showed that administration of PPE dose‐dependently decreased the MDA level, which represents the suppression of oxidative stress. We observed a dose‐dependent increase in thiol and SOD levels following PPE treatment. The extract also dose dependently reduced AchE in both hippocampus and cortex. Several in vitro experiments have indicated the antioxidant, free radical scavenging, and AchE inhibitory potential of pomegranate peel extracts (Chasanah, 2020; Morzelle et al., 2019; Derakhshan et al., 2018). Interestingly, these effects were linked to phenolic compounds of pomegranate peel extracts (Chasanah, 2020; Eddebbagh et al., 2016). An ethanolic extract of pomegranate peel was reported to contain phenolic compounds ranging from 276 to 413 mg of gallic acid equivalent/extract (g) (Derakhshan et al., 2018). According to recent studies, PPE seems to protect against oxidative stress through the upregulation of antioxidant proteins (Ramadan & Alkarim, 2021). In this context, ellagic acid, a polyphenolic compound present in PPE, modulated oxidative stress as indicated by increasing SOD, glutathione, total antioxidant capacity in serum, and SOD gene expression, while decreasing MDA in the brain of AlCl_3_‐injured rats. Furthermore, it attenuated Aβ toxicity and apoptosis associated with the improvement of episodic memory using the novel object recognition test (Ramadan & Alkarim, 2021). Morzelle et al. (2016) showed that supplementation of PPE (800 mg/kg) alleviated neuroinflammation and oxidative injury in Aβ‐challenged mice. Moreover, PPE ameliorated spatial memory along with reduced amyloid plaque density and AchE activity (Morzelle et al., 2016). Another study also demonstrated the antioxidant and inflammatory potential of the methanolic extract of *P. granatum* peel as indicated by the suppressed nitric oxide, TNF‐α, and IL‐6 levels in lipopolysaccharides stimulated microglial BV2 cells (Syafii et al., 2021). Accordingly, the inhibitory effects of PPE on oxidative stress and AchE activity might explain the basic mechanisms underlying our behavioral observations.

To further investigate the neuroprotective mechanisms of PPE against scopolamine brain injury, Nrf2 and HO‐1 mRNA expression were examined in this study. Scopolamine‐induced cognitive decline was associated with Nrf2‐HO‐1 downregulation (Lee et al., 2017; Tang, 2019), as we observed in our research. Some evidence indicates that pomegranate extract and ellagic acid counteracted hepatotoxicity and neurotoxicity by activating Nrf2‐HO‐1 (Baluchnejadmojarad et al., 2017; Husain et al., 2018; Li et al., 2017). In this context, ellagic acid exerted neuroprotection via upregulating Nrf2‐HO‐1 expression in neurotoxin oxidopamine‐mediated Parkinson's disease (Baluchnejadmojarad et al., 2017). In support of the previous results, our research showed that PPE elevated Nrf2 and HO‐1 mRNA expression in the hippocampus.

According to the findings of current research, PPE could be recommended for further investigation in AD management. We showed that 400 and 800 mg/kg of PPE were effective against memory and learning dysfunction. The doses of PPE administration were selected based on previous studies (Akbarian et al., 2022; Belkacem et al., 2010; Sarkaki et al., 2015). LD_50_ of the pomegranate peel extract was reported to be over 2000 mg/kg in rodents (Ahad et al., 2018). Hence, PPE seems to be safe at the doses which were studied in the current study. Although effective doses of PPE for clinical application were not assessed in the current research, it was previously shown that PPE supplementation could be well tolerated and safe at doses as high as 6000 mg/day in humans (Ahad et al., 2018; Kamali et al., 2015). Considering the previous evidence (Reagan‐Shaw et al., 2008), the human‐equivalent doses of PPE in the present study seem to be within the safe range. However, additional research should be conducted to confirm the antiamnesic efficacy of PPE in patients with dementia disorders.

## 5 CONCLUSION

According to our data, the preventive effect of PPE on scopolamine‐induced cognitive impairment might be associated with attenuation of oxidative stress damage via up‐regulation of Nrf2‐HO‐1 in brain tissue.

## CONFLICT OF INTERESTS

The authors did not declare any conflict of interest.

## Data Availability

The datasets used and/or analyzed during the current study are available from the corresponding author on reasonable request
